# Clinical and Genetic Profiles of 11 Chinese Patients With Angelman Syndrome

**DOI:** 10.1155/genr/5593007

**Published:** 2025-12-05

**Authors:** Song Qu, Pu Sun, Limeng Dai, Cui Song, Yanyan Wang

**Affiliations:** ^1^ Department of Medical Genetics, College of Basic Medical Science, Army Medical University (Third Military Medical University), Chongqing, China, tmmu.edu.cn; ^2^ Maternal and Child Health Hospital of Yongchuan District, Chongqing, China; ^3^ Department of Gynecology and Obstetrics, Southwest Hospital, Army Medical University (Third Military Medical University), Chongqing, China, tmmu.edu.cn; ^4^ National Clinical Research Center for Child Health and Disorders, Ministry of Education Key Laboratory of Child Development and Disorders, Chongqing Key Laboratory of Pediatrics, Children’s Hospital of Chongqing Medical University, Chongqing, China, chcmu.com

**Keywords:** Angelman syndrome, genotype, phenotype

## Abstract

Angelman syndrome (AS) is a severe neurodevelopmental disorder resulting from different molecular mechanisms. Investigating the correlation between genotypes and phenotypes is crucial to facilitate accurate diagnosis and effective prevention strategies for this disorder. However, determining the genotypes of patients to analyze genotype‒phenotype correlations is challenging when parental genetic information is lacking. Therefore, we proposed a genotyping strategy for use with 11 unrelated Chinese patients with AS who were recruited for this study. The strategy involved a combination of methylation‐specific polymerase chain reaction (MS‐PCR), exome sequencing (ES), Sanger sequencing, and MS multiplexed ligation probe amplification (MS‐MLPA). The results revealed that the number of molecular deletions involving the critical 15q11. 2‐q13 region (54.5%) was lower than that reported in other studies of Chinese patients. In addition, the prevalence of patients with imprinting defects (IDs) (27.3%) and variants (18.2%) was greater, whereas the proportion of patients with uniparental disomy (UPDs) was lower. We also summarized the characteristics of patients with different genotypes and analyzed the correlations between genotypes and phenotypes. Compared with the consensus for diagnostic criteria, our results showed that several features were less common, including the combination of frequent laughing/smiling, tremulous limb movements, ataxia of gait, and microcephaly. Conversely, the incidence of both epilepsy and abnormal electroencephalograms (EEGs) was greater. Notably, a novel mutation in the *UBE3A* gene that had not been previously reported was identified in a family.

## 1. Introduction

Angelman syndrome (AS) (OMIM: #105830) is a rare neurodevelopmental disorder, with an estimated prevalence ranging from 1/20,000 to 1/12,000. It results from loss of function of the imprinted ubiquitin–protein ligase E3A (*UBE3A*) gene on Chromosome 15q11.2–q13 [1]. *Ube3a* is biallelically expressed in most tissues of the body; however, in rodents and humans, because of genomic imprinting, most neurons express *Ube3a* only from the maternally inherited allele; the paternal allele of *UBE3A* is epigenetically silenced (i.e., paternally imprinted). Maternal inactivation of *UBE3A* causes nearly complete loss of UBE3A protein selectively from the brain [2].

Because molecular mechanisms determine the recurrence risk, prognosis, and clinical phenotypes, understanding the associated molecular mechanisms and genetic profiles can help clinicians make an accurate diagnosis and provide appropriate genetic counseling to patients and their families. The study of genotypes and phenotypes is important for establishing consensus and deepening the understanding of genetic profiles.

The genotypes are classified into four categories on the basis of their molecular mechanisms: (1) 15q11. 2‐q13 deletion in 70%–75% of patients, (2) variant in the *UBE3A* gene in 10%–15% of patients, (3) paternal uniparental disomy (UPD) in 1%–3% of patients, and (4) imprinting defect (ID) in 2%–4% of patients. Additionally, 10% of patients present with clinical characteristics of AS, yet the underlying genetic cause remains undetermined [2]. Many studies on genotype‒phenotype correlations have shown that patients with deletions have the most typical and severe phenotypes, such as developmental delay and intellectual disability [3–5]. Patients with UPDs and IDs exhibit considerably reduced incidences of hypopigmentation, microcephaly, and severe epilepsy. Furthermore, patients with *UBE3A* variants present characteristics of intermediate severity. The incidences of epilepsy, speech impairment, and microcephaly are statistically similar to that reported in patients with deletions [3–6]. These researchers genotyped patients on the basis of an analysis of the parental origin of chromosomes. Given that AS is a genetically imprinted disease, it is essential to determine the origin of chromosomes by analyzing the genetic information of patients’ parents. However, many patients were excluded from previous studies because their parents’ blood samples were not collected, which reduced the sample size.

To address this limitation, we proposed and designed a genotyping strategy that used exome sequencing (ES), methylation‐specific multiplexed ligation probe amplification (MS‐MLPA), and Sanger sequencing to identify genotypes for patients without parental blood samples. ES is a powerful tool that can analyze a range of genetic variations, including copy number variations (CNVs), single‐nucleotide variants (SNVs), and insertions/deletions (InDels). Furthermore, it can be used to evaluate chromosomal heterozygosity through the examination of SNVs and InDels. Moreover, MS‐MLPA is a highly specific technique that enables the examination of gene copy numbers and methylation levels. Thus, combining ES, MS‐MLPA, and Sanger sequencing has the potential to genotype patients whose parental blood is unavailable and increase study sample sizes.

In this study, we retrospectively investigated the clinical characteristics of 11 Chinese children with AS. We first genotyped these patients without parental blood samples using our genotyping strategy. We subsequently investigated the genetic profiles and correlations between genotypes and clinical phenotypes. In addition, we reported a family line carrying a novel variant that enriches the spectrum of disease variants in AS.

## 2. Materials and Methods

### 2.1. Patients

We recruited 11 patients with AS who were admitted to the Children’s Hospital of Chongqing Medical University from March 2007 to July 2018, including 4 males (36.4%) and 7 females (63.6%); of these patients, 8 were 5–8 years old, 2 were 10–11 years old, and 1 was 22 years old. Informed consent or assent was obtained from the patients or their parents, respectively, when blood samples were collected in EDTA anticoagulant tubes. All participants were clinically diagnosed with AS and methylation‐specific polymerase chain reaction (MS‐PCR) was performed. The results revealed that 9 participants were positive and 2 were negative in terms of their MS‐PCR. This study was approved by the Ethics Committee of the Children’s Hospital of Chongqing Medical University (Ethics Committee Approval No. [2021] Ethics [Research] No. [279]).

### 2.2. Symptom Data Collection

A scale based on the clinical phenotypic features of the AS diagnostic criteria was developed to collect basic information on and the clinical features of the patients, including common features, such as intellectual disability, developmental delay, speech impairment, a combination of frequent laughing/smiling, tremulous limb movement, and gait ataxia. In addition, three symptoms were identified as common: epilepsy, abnormal electroencephalograms (EEGs), and microcephaly. Moreover, the following symptoms were identified as being associated with AS: sleep disturbance, prognathia, protruding tongue, sucking/swallowing difficulties, frequent drooling, constipation, obesity, hypopigmented skin, and scoliosis.

### 2.3. DNA Extraction

Two milliliters of blood were collected from each patient’s median elbow vein and anticoagulated with EDTA‐K2, and genomic DNA was extracted via a whole‐blood genomic DNA extraction kit (Tiangen, China).

### 2.4. MS‐PCR

MS‐PCR was employed to assess the methylation status of the *SNRPN* gene, with primers and PCR conditions as described previously [7].

### 2.5. ES

Genomic DNA was extracted from each patient’s peripheral blood and subsequently purified and sorted. The DNA library was then constructed via the SureSelectXT Reagent Kit, and all exons were captured via the SureSelectXT Human All Exon Kit V6 probe, followed by the hybridization of the library. The captured DNA fragments were sequenced on the Illumina HiSeq/NovaSeq platform (Illumina, USA) in 2 × 150 bp double‐ended sequencing mode for high‐throughput sequencing, resulting in FASTQ data. EXCAVATOR2 software (https://sourceforge.net/projects/excavator2tool/) was used to identify CNVs, whereas the GATK HaplotypeCaller method was employed to detect SNVs/InDels within each sample. The identified variants were then filtered according to the screening scheme recommended by the software.

### 2.6. MS‐MLPA

A Prader–Willi/AS MS‐MLPA kit (MRC‐Holland, The Netherlands, ME028‐C1) was used in accordance with the standard protocol. Two hundred nanograms of genomic DNA were denatured at 98°C for five minutes and subsequently hybridized to the ME028 probe mixture for 16 h at 60°C. The probe covered the regions encompassing the critical regions of u1b, intr. u2, u5, intr. 2, intr. 3, and intr. 7 of the *SNRPN* gene (NM_022807.5) and Exons 1–5, Exon 10 of the *UBE3A* gene (NM_130838.4), and Exon 1 of the *MAGEL2* gene (NM_019066.5). The products were separated into two distinct tubes: one designated for copy number analysis and the other for methylation analysis using methylation‐sensitive endonucleases. The PCR products were analyzed using an ABI 3130xl Genetic Analyzer (Applied Biosystems, Foster City, CA, USA), and the resulting data were analyzed via GeneMark v.1.51 (SoftGenetics, State College, PA, USA). The peak intensity was normalized through internal control probes, and the intensity ratios of the same probes in the samples were compared with those of the controls.

### 2.7. Sanger Sequencing

**Figure 1:**
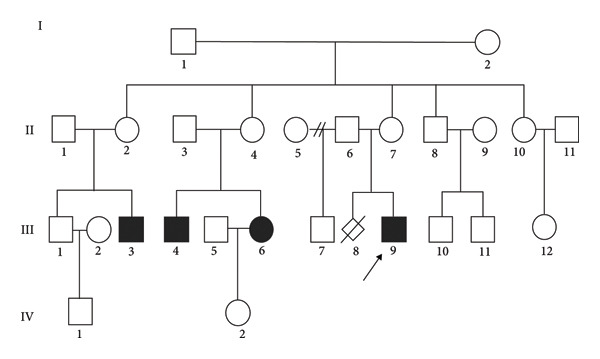
Pedigree map of the family lineage with the c.2369_2374delinsGGA variant. Notably, the symbols □ and ○ represent normal males and females, respectively, whereas the symbols ■ and ● represent diseased males and females, respectively. III‐9 is the proband, whereas III‐3, 4, and 6 are patients investigated from the family lineage, which presents a genetic imprinting mode of inheritance (paternal imprinting and maternal expression).

Primers were designed using Primer Premier 5 software based on the *UBE3A* gene sequence (NC000015.10) to amplify the DNA fragments containing suspected *UBE3A* variants identified through ES: c.2369_2374delinsGGA (p.Asp790_Arg791delinsGly) (ClinVar: SCV006307961) for Patient 1 and c.2556_2665del (p.L852fs) for Patient 2. The PCR primer sequences for two patients with suspected *UBE3A* variants were *UBE3A*_c.2369delins for Patient 1 (forward primer: 5′‐CAG​TTC​ATA​TGT​ATG​TGA​CGA​GGA​A‐3′; reverse primer: 5′‐ACTTCCAA GTT​GTC​TCT​TAA​TAT​AG‐3′) and *UBE3A*_c.2556del for Patient 2 (forward primer: 5′‐GTA​TTT​CCC​ATG​ACT​TAC​AGT‐3′; reverse primer: 5′‐CCA​ATA​AAG​AAG​GGA​GGC‐3′). Family members of the patients were subjected to Sanger sequencing to determine these *UBE3A* variants. For Patient 1, nine family members were tested, namely, I‐1, I‐2, II‐1, II‐2, II‐6, II‐7, III‐1, III‐4, and III‐9 (Figure 1). Patient 2, his father, and mother were included in Sanger sequencing. The genomic regions where candidate pathogenic *UBE3A* variants are located were amplified, and the resulting PCR products were purified. The sequencing reactions were subsequently performed through the BigDye Terminator v3.1 sequencing kit (Thermo Fisher Scientific, USA), and the products were assayed on an ABI 3730XL genetic analyzer (Thermo Fisher Scientific, USA).

### 2.8. Bioinformatic Analysis

The conservation of amino acid residues and the pathogenicity of the variants were predicted via several in silico analysis techniques [8]. PhyloP, PhastCons, SnapGene, PROVEAN, and MutationTaster2 were used to predict the effects of variants on protein function, whereas SWISS MODEL was used to predict the effects of variants on protein structure.

## 3. Results

### 3.1. ES

In 6 of the 9 MS‐PCR‐positive patients, the genotype of the 15q11.2‐q13 deletion was identified via ES. In the remaining 3 cases, which may have been genotyped as ID or UPD, no suspected pathogenic variants or copy number deletions were identified. As shown in Supporting Table 1, analysis of Chromosome 15 loci revealed a heterozygous background across all subjects. However, since only a single *UBE3A* variant was reported and the percentages of the heterozygous variants were less than 40%, segmental UPD cannot be excluded without STR marker analysis. In addition, the ES identified potentially pathogenic variant sites in the *UBE3A* gene in 2 MS‐PCR‐negative patients. Therefore, these two families should undergo Sanger sequencing with primers designed to amplify the relevant fragment.

### 3.2. MS‐MLPA

**Figure 2:**
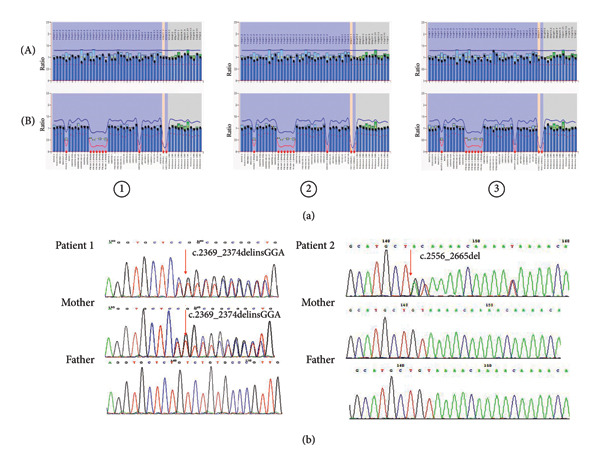
Results of the MS‐MLPA and Sanger sequence. (a) Patients ①, ②, and ③, who had ES‐negative results, showed maternal aberrant demethylation of the imprinting center through MS‐MLPA. The copy number results for 15q11.2‐q13 are presented in Panel A, whereas Panel B shows the CNV results following methylation‐sensitive enzymatic cleavage. (b) DNA sequence chromatograms of c.2369_2374delinsGGA (a) and c.2556_2665del (b) in *UBE3A* (NM_130838.4) from patients and their parents.

The MS‐MLPA was applied to three patients who had undergone ES and were negative for abnormalities. The MS‐MLPA results showed aberrant methylation of u1b, intr.u2, u5, intr.2, intr.3, and intr.7 of the *SNRPN* gene (NM_022807.5) and Exon 1 of the *MAGEL2* gene (NM_019066.5) (Figure 2(a)). The normal result for this region is maternal methylation. However, the results revealed aberrant maternal demethylation, and the corresponding genomic sequences with deletions were not identified. Since our methodology cannot distinguish between ID and UPD, a haplotype analysis should be performed to support the ID genotype. Owing to the rarity of heterodisomy [9, 10] and the results of the ES, these cases are classified as ID in this study. Because UPD cannot be ruled out, the limitations of this classification are discussed in Section 4.1: Discussion of the manuscript.

### 3.3. Sanger Sequencing

Sanger sequencing maps of the 2 mutant patients and their parents revealed the presence of a previously unreported variant in the *UBE3A* gene (NM_130838.4), specifically c.2369_2374delinsGGA (p.Asp790_Arg791delinsGly). Both Patient 1 and his mother had the c.2369_2374delinsGGA variant, whereas the father had no variants at this locus (Figure 2(b)). These findings indicate maternal inheritance of the variant in Patient 1. Importantly, a pedigree analysis combined with genotyping of extended family members (Table 1) suggested that this variant likely arose from a de novo mutation in the maternal grandfather (I‐1), as evidenced by the following: (1) absence of the variant in the maternal grandmother (I‐2), (2) its presence in the proband’s unaffected mother (II‐7) and maternal aunt (II‐2), and (3) its transmission to affected grandchildren exclusively through carrier females. The results of Sanger sequencing of the family members (I‐1, I‐2, II‐6, II‐7, II‐1, II‐2, III‐1, III‐4, and III‐9) of Patient 1 are summarized in Table 1. Patient 2 was characterized by the presence of the c.2556_2665del (p.L852fs) variant in the *UBE3A* gene, whereas the proband’s mother did not carry this variant. Therefore, the variant observed in this patient was de novo. The c.2556_2665del (p.L852fs) variant in the *UBE3A* gene has been previously reported [11].

**Table 1:** Sequencing results of the family members of Patient 1.

Sample name	Sequencing results
I‐1	exon9 c.2369_2374delinsGGA
I‐2	Normal
II‐1	Normal
II‐2	exon9 c.2369_2374delinsGGA
II‐6	Normal
II‐7	exon9 c.2369_2374delinsGGA
III‐1	Normal
III‐4	exon9 c.2369_2374delinsGGA
III‐9	exon9 c.2369_2374delinsGGA

*Note:* The sample names (I‐1, I‐2, II‐1, II‐2, II‐6, II‐7, III‐1, III‐4, and III‐9) were derived from the pedigree map of the c.2369_2374delinsGGA mutation family lineage (Figure 1).

### 3.4. Phenotypic Difference Analysis of Patients With Different Genotypes

**Figure 3:**
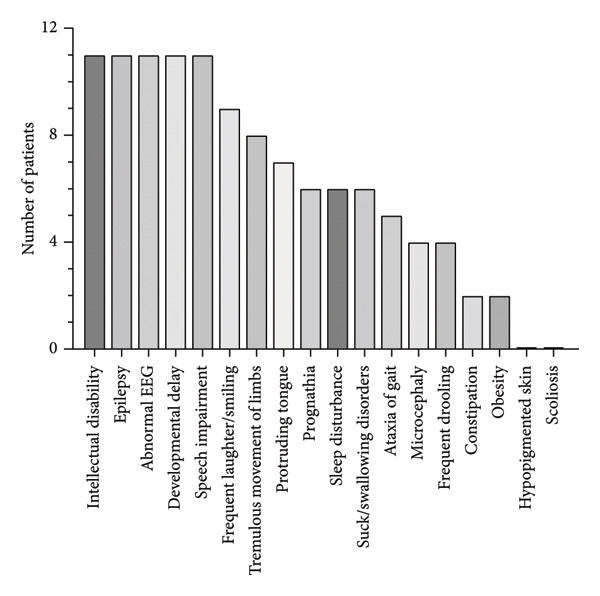
Summary of the clinical phenotypes of AS (*n* = 11). The frequency of the main clinical features defined in consensus diagnostic criteria was counted among the patients. These clinical features included intellectual disability, epilepsy, abnormal electroencephalograms, developmental delays, and language disorders.

Compared with AS patients in China [3, 4], the proportion of patients with a deletion (6/11, 54.5%) was lower, incidences of patients with IDs (3/11, 27.3%) and variant (2/11, 18.2%) were greater, and the proportion of UPD was lower. According to the international diagnostic standards of AS, the clinical characteristics of patients are divided into consistent, frequent, and associated features [10]. In our study, compared with the consensus for diagnostic criteria of AS [10], these clinical features (the combination of frequent laughing/smiling, tremulous limb movement, gait ataxia, microcephaly, constipation, obesity, and hypopigmented skin) were lower than consensus (Table 2). A statistical summary of the clinical manifestations observed in all patients with AS is presented (Figure 3). Despite some features being less common in our cohort, the core symptoms (intellectual disability, developmental delay, speech impairment, epilepsy, and abnormal EEGs) were universally present, which is consistent with the international consensus.

**Table 2:** Summary of the clinical phenotypic classification of AS.

Clinical features	Genotypes			Average
	Deletion	Mutation	ID	
Consistent (100%)				
Intellectual disability	6/6	2/2	3/3	100.00%
Developmental delay	6/6	2/2	3/3	100.00%
Speech impairment	6/6	2/2	3/3	100.00%
Combination of frequent laughter/smiling	6/6	2/2	1/3	81.82%
Tremulous limb movement	5/6	1/2	2/3	72.73%
Gait ataxia	3/6	1/2	1/3	54.55%
Frequent (more than 80%)				
Epilepsy	6/6	2/2	3/3	100.00%
Abnormal EEG	6/6	2/2	3/3	100.00%
Microcephaly	2/6	1/2	1/3	54.55%
Associated (20%–80%)				
Sleep disturbance	4/6	1/2	1/3	54.55%
Prognathia	4/6	1/2	1/3	54.55%
Protruding tongue	3/6	1/2	3/3	63.64%
Suck/swallowing disorders	3/6	1/2	2/3	54.55%
Frequent drooling	2/6	1/2	1/3	36.36%
Constipation	1/6	1/2	0/3	18.18%
Obesity	1/6	1/2	0/3	18.18%
Hypopigmented skin	0/6	0/2	0/3	0.00%
Scoliosis	0/6	0/2	0/3	0.00%
Average	59.26%	61.11%	51.85%	57.41%

*Note:* Williams et al. proposed a classification of AS symptoms on the basis of the percentage of symptoms observed clinically in 2006: consistent (100%), frequent (80%), or associated (20%–80%).

### 3.5. A Novel Variant in UBE3A in a Family

Because the c.2369_2374delinsGGA variant has not been previously reported, we investigated the pathogenicity of the variant and the inheritance pattern of the family. Following comprehensive genetic counseling, physical examination, genetic testing, and analysis of imaging and diagnostic data, the members of this family were evaluated. We constructed a detailed four‐generation pedigree (Figure 1) comprising 26 individuals. The investigation revealed that the disease is inherited within this lineage in a gene‐imprinting manner, specifically paternal imprinting and maternal expression. This information is extremely important, as it provides invaluable insight into the mechanisms underlying the *UBE3A* gene variant and AS.

**Figure 4:**
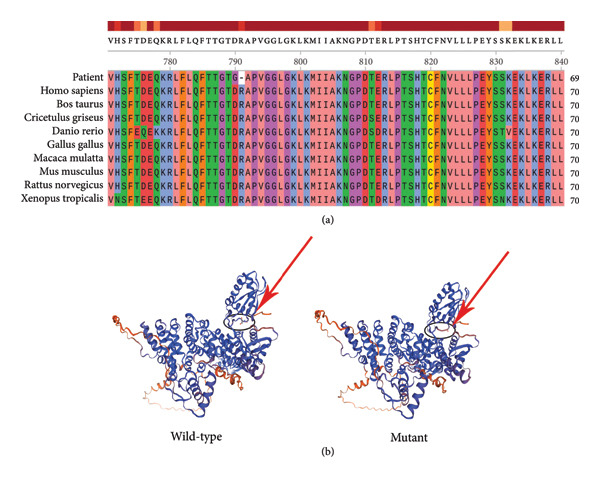
Alignment and protein virtual construction for the c.2369_2374delinsGGA (p.Asp790_Arg791delinsGly) variant. (a) Alignment of the p.Asp790_Arg791delinsGly variant with UBE3A orthologs in different vertebrate species. (b) Structural model diagrams of *UBE3A*‐encoded proteins. (a) The structural model of the wild‐type *UBE3A*‐encoded protein; (b) the structural model of the protein encoded by *UBE3A* with the c.2369_2374delinsGGA variant. The UBE3A protein model in (b) exhibited a reduction in β‐folding at two regions in comparison to that of the wild type.

The conservation of amino acid residues and pathogenicity of c.2369_2374delinsGGA (p.Asp790_Arg791delinsGly) were predicted via several in silico analysis techniques. PhyloP [9] and PhastCons [12] demonstrated that the amino acid residues were highly evolutionarily constrained (Table 3). Amino acid sequence alignment showed that the p.Asp790_Arg791delinsGly variant occurs as a highly conserved residue in UBE3A, with surrounding amino acid residues conserved between orthologs (Figure 4(a)). PROVEAN [13] and MutationTaster2 [14] revealed that the functional effect of the p.Asp790_Arg791delinsGly variant was deleterious (Table 3). In addition, the UBE3A protein model with the p.Asp790_Arg791delinsGly variant presented reduced β‐folding in two regions compared with the wild type, as revealed by the virtual protein construction system (Figure 4(b)).

**Table 3:** Conservation of amino acid residues and pathogenicity of the *UBE3A* gene c.2369_2374delinsGGA (p.Asp790_Arg791delinsGly) mutation as predicted by multiple in silico analysis techniques.

Method	Score	Prediction
PhyloP	6.095	Conserved
PhastCons	1.000	Conserved
Mutation taster	1.000	Deleterious
PROVEAN	−19.027	Deleterious

## 4. Discussion

AS is a neurodevelopmental disorder that is characterized by severe intellectual disability, speech deficits, a happy personality, ataxia, epilepsy, and unique behavioral features [1]. In this study, we first genotyped these patients in the absence of parental blood samples using our genotyping strategy. We subsequently investigated the genetic profiles and correlations between genotypes and clinical phenotypes. Finally, we reported a family line carrying a novel variant that enriches the spectrum of disease variants in AS.

### 4.1. Limitation of Our Genotyping Strategy

**Figure 5:**
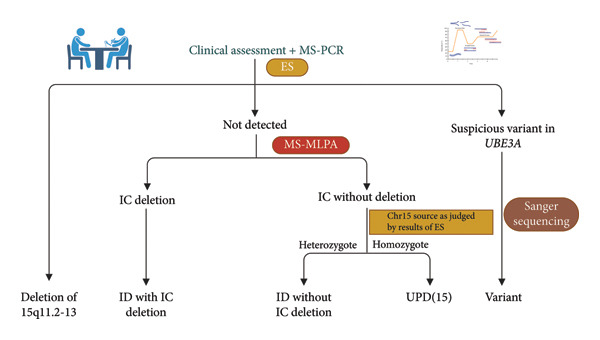
Genotyping strategy for AS patients. Initially, WES was used to identify the genotypes of patients with deletions and variants. Sanger sequencing was subsequently applied to validate the suspected variant. Finally, MS‐MLPA was used in combination with the ES results to determine the genotypes of patients with ES‐negative results.

As MS‐PCR provides a diagnosis in 80% of the cases except for sequence variants, chromosomal microarray (CMA) and MS‐MLPA are usually used to test for deletions, UPD, and ID. Our strategy to employ ES for both MS‐PCR‐positive and MS‐PCR‐negative cases was a choice made primarily to overcome the fundamental challenge of unavailable parental samples, and ES enabled molecular subtyping and provided comprehensive genetic profiling. Although our genotyping strategy has the potential to genotype patients whose parental blood samples are unavailable, there are still areas for improvement. In this study, we genotyped the parental blood of 11 unrelated Chinese patients via a genotyping strategy (Figure 5). The genotyping results revealed that 6 patients had deletions, 3 had IDs, and 2 had variants.

However, the ability of this study to distinguish ID from UPD is limited. It is difficult to accurately determine the origin of chromosomes in the absence of parental information. With our strategy, the detection of CNVs, SNVs, and InDels via ES was used to determine the origin of two Chromosome 15s and to differentiate patients with ID from those with UPD. However, UPD is complicated and can be divided into 3 types: (1) isodisomy, (2) heterodisomy, and (3) partial heterodisomy and partial isodisomy. Patients with heterodisomy and ID have the same results according to ES. Therefore, the ID patients we genotyped may have included patients with heterodisomy. Fortunately, the possibility of heterodisomy is extremely low [15].

To improve the accuracy of differentiating ID from UPD, particularly for cases with heterodisomy or mixed hetero/isodisomy, complementary approaches such as CMA and STR marker analysis of Chromosome 15 could be employed. CMA provides high‐resolution detection of copy‐neutral regions of homozygosity (ROH) that may indicate uniparental isodisomy [16]. Moreover, STR analysis of polymorphic markers along with Chromosome 15 can be used to definitively trace parental chromosomal origin by assessing haplotype transmission patterns [17]. These methods are specifically recommended in the EMQN/ACGS guidelines when parental samples are available. Our strategy is specifically designed for scenarios where parental samples are unavailable, which is a common limitation of retrospective studies. However, analysis of parental genetic information remains the gold standard for definitive genotyping, as recommended by the EMQN/ACGS guidelines [17]. Deletions, UPDs, and IDs can be identified through the detection of hypomethylation or a complete absence of methylation in patients and their parents. However, microsatellite analysis must be used to examine chromosomal origin when this approach is used.

### 4.2. Clinical and Genetic Profiles of AS Patients

In our study, 11 children with clinically confirmed AS were included. A total of 54.5% of these mutations were caused by deletions, 27.3% by IDs, and 18.2% by variants in the *UBE3A* gene. The prevalence of genotypes differed from that in previous reports [3, 4]. In Western populations, approximately 70%–75% of AS events are caused by deletions, 2%–7% by UPDs, 3%–5% by IDs, and approximately 10% by variants. In a large cohort of Chinese children, 83.02% of AS cases were caused by deletions, 4.46% by UPDs, 1.44% by IDs, and approximately 9.35% by variants. While the small sample size and limitations of our genotyping strategy may contribute to these differences, the observed distribution also suggests potential population‐specific differences in the genetic architecture of AS among Chinese patients, which warrants further investigation in larger cohorts. In particular, higher prevalences of IDs and variants were observed, as well as relatively lower prevalences of deletions and UPDs.

Our findings support the conclusions of many genotype‒phenotype correlation studies and demonstrate that patients with deletions have a greater number of clinical features and a more severe phenotype [3–6]. Conversely, patients with IDs have the mildest phenotype. Our phenotype analysis showed that patients with IDs had the lowest percentage (51.85%) of the clinical features listed in Table 2. Patients with variants had the highest percentage (61.40%) of clinical features, but the patients with variants had the mildest symptoms. In contrast, patients with deletions accounted for only 59.26% but had the most severe symptoms. We also found that patients with the same genotype presented different clinical profiles and severity levels. We confirm that larger deletions (> 5 Mb) consistently show severe manifestations due to the loss of critical genes (including *GABRB3/GABRA5*), which aligns with established genotype–phenotype relationships. One of the patients with ID presented more severe symptoms than many patients with deletions did, whereas a patient with a deletion also presented milder symptoms. We agree that phenotypic variability within the deletion genotype is significantly influenced by specific genomic structural differences, such as the deletion boundaries, the size, and the involvement of key genes (Supporting Table 2). Among three patients with identical 3.4 Mb deletions (Cases 04/05/06), Patient 6 exhibited markedly milder symptoms, particularly in seizure frequency and severity. We attribute this variability to (1) early intensive intervention in the therapeutic window, (2) potential individual differences in epigenetic regulation, and (3) genetic background (such as modifier genes).

In addition, the prevalences of some clinical features (intellectual disability, developmental delay, speech impairment, epilepsy, and abnormal EEG) in our study were not consistent with consensus. It can be concluded that consensus for the Western population may not always be suitable for the Chinese population. Therefore, it is essential to develop consensus that is consistent with the clinical profiles of Chinese patients [2, 4].

### 4.3. Research on the Function of the Novel Variant

We conducted a comprehensive bioinformatic analysis to predict the pathogenicity of the variant in our study, but the function of the p.Asp790_Arg791delinsGly variant in UBE3A is unknown. Bioinformatic analysis revealed that the amino acid residues were highly evolutionarily constrained and that the variant was deleterious. In addition, the variant occurred in the primary region of the HECT structural domain of UBE3A, which is responsible for substrate recognition. The deletion of this region may prevent the formation of ubiquitin thioester bonds, thereby affecting the process of protein ubiquitylation [18]. However, the role of this deletion in the abnormal function of UBE3A and the underlying mechanism of how it causes AS remain unclear. Exploring how this variant affects the function of UBE3A and leads to AS is extremely important for understanding the underlying mechanisms of this disorder. In future studies, we can model mice with this variant and explore the underlying mechanism through behavioral tests and differential expression profiles.

## 5. Conclusions

The distribution of genotypes in this study of a small number of AS individuals differed from that reported in larger sized populations. It is important to establish a suitable regional consensus to inform clinical practice. Suitable consensus and genotyping can help inform genetic counseling and intervention. This novel variant not only expands the range of AS variants but also has significant potential to enhance our understanding of the underlying mechanisms and inheritance patterns of AS.

## Ethics Statement

This study was approved by the Ethics Committee of the Children’s Hospital of Chongqing Medical University (Ethics Committee Approval No. [2021] Ethics [Research] No. [279]).

## Consent

Informed consent was obtained from the parents of the patients.

## Conflicts of Interest

The authors declare no conflicts of interest.

## Author Contributions

Song Qu: patient genetic typing, data analysis, and article writing; Pu Sun: case collection; Limeng Dai: experimental design; Cui Song: research guidance and phenotype collection; Yanyan Wang: study design, research guidance, data analysis, article writing, and financial support.

## Funding

This work was supported by the grants from the National Natural Science Foundation of China (82171852), Chongqing Science and Technology Commission (cstc2021jcyj‐msxmX0329), and the Key Project of Innovation and Development United Fund of Chongqing Natural Science Foundation (CSTB2022NSCQ‐LZX0029).

## Supporting Information

Additional supporting information can be found online in the Supporting Information section.

## Supporting information


**Supporting Information 1** Supporting Table 1: List of annotated variants on Chromosome 15 identified by whole‐exome sequencing.

**Supporting Information 2** Supporting Table 2: Correlation between 15q11.2‐q13 deletion sizes and phenotypic variability in patients with Angelman syndrome.

## Data Availability

The datasets generated and/or analyzed during the current study are available from the corresponding author upon reasonable request.
