# Supplementary material for: Clinical and Genetic Profiles of 11 Chinese Patients With Angelman Syndrome
Source: Genet Res (Camb). 2025 Dec 5;2025:5593007. doi: 10.1155/genr/5593007 (PMC12721727; doi:10.1155/genr/5593007)
Supplement: Supplementary file 2 — Supporting Information 2 Supporting Table 2: Correlation between 15q11.2‐q13 deletion sizes and phenotypic variability in patients with Angelman syndrome. [file GENR-2025-5593007-s002.docx]

**Supplementary Table 2** Correlation Between 15q11.2-q13 Deletion Sizes and Phenotypic Variability in patients with Angelman syndrome

| Patient ID | 1 | 2 | 3 | 4 | 5 | 6 |
| --- | --- | --- | --- | --- | --- | --- |
| Chromosomal coordinates (hg38) | chr15: 20,739,496-28,775,614 | chr15: 23,599,879-28,778,209 | chr15: 22,646,194-26,174,061 | chr15: 25,200,059-28,603,217 | chr15: 25,200,059-28,544,836 | chr15: 25,200,059-28,594,302 |
| Deletion Size (bp) | 8,036,118 | 5,178,336 | 3,527,868 | 3,403,158 | 3,344,777 | 3,394,243 |
| Key genes affected | *UBE3A, GABRB3, GABRA5, OCA2* | *UBE3A, GABRB3, GABRA5* | *UBE3A, ATP10A* | *UBE3A, GABRG3* | *UBE3A, GABRG3* | *UBE3A, GABRG3* |
| Intellectual disability | **+** | **+** | **+** | **+** | **+** | **+** |
| Developmental delay | **+** | **+** | **+** | **+** | **+** | **+** |
| Speech impairment | **+** | **+** | **+** | **+** | **+** | **+** |
| Combination of frequent laughter/smiling | **+** | **+** | **+** | **+** | **+** | **+** |
| Tremulous limb movement | **+** | **+** | **+** | **+** | **+** | **-** |
| Gait ataxia | **+** | **+** | **+** | **-** | **-** | **-** |
| Epilepsy | **+** | **+** | **+** | **+** | **+** | **+** |
| Abnormal EEG | **+** | **+** | **+** | **+** | **+** | **+** |
| Microcephaly | **-** | **-** | **-** | **+** | **+** | **-** |
| Sleep disturbance | **+** | **+** | **+** | **-** | **-** | **+** |
| Prognathia | **+** | **+** | **+** | **+** | **-** | **-** |
| Protruding tongue | **+** | **-** | **+** | **-** | **+** | **-** |
| Suck/swallowing disorders | **+** | **-** | **-** | **+** | **+** | **-** |
| Frequent drooling | **-** | **-** | **+** | **+** | **-** | **-** |
| Constipation | **+** | **-** | **-** | **-** | **-** | **-** |
| Obesity | **-** | **+** | **-** | **-** | **-** | **-** |
| Hypopigmented skin | **-** | **-** | **-** | **-** | **-** | **-** |
| Scoliosis | **-** | **-** | **-** | **-** | **-** | **-** |
| Clinical severity | **+++** | **+++** | **++** | **++** | **++** | **+** |

Genomic coordinates are based on the GRCh38/hg38 assembly. Symbols "+" and "-" denote presence or absence of clinical features, respectively. Clinical severity was categorized as: severe (+++), moderate (++), or mild (+).
